# Development and Validation of a Decision-Making Stratification Algorithm to Optimize the Use of Rapid Diagnostic Testing for Patients with* Staphylococcus* Bacteremia

**DOI:** 10.1155/2017/8648137

**Published:** 2017-05-30

**Authors:** Thamer A. Almangour, Abdullah A. Alhifany, Deanne E. Tabb

**Affiliations:** ^1^Department of Pharmacotherapy and Experimental Therapeutics, University of North Carolina at Chapel Hill, Eshelman School of Pharmacy, Chapel Hill, NC, USA; ^2^Department of Pharmacy Practice and Science, University of Arizona College of Pharmacy, Tucson, AZ, USA; ^3^Columbus Regional Health, Midtown Medical Center, Columbus, GA, USA

## Abstract

**Purpose:**

To evaluate whether introducing rapid diagnostic testing in conjunction with implementing a stratification algorithm for testing eligibility would be an appropriate clinical and cost saving approach.

**Method:**

An internal concurrent 4-month observational study was performed. Positive blood cultures continued to be worked up in accordance with standard of care. An additional call to the infectious disease (ID) pharmacy service occurred for all positive blood cultures with Gram-positive cocci in clusters (GPCC). The ID pharmacy service investigated each case using a prespecified stratification algorithm to minimize unnecessary use of rapid identification testing.

**Results:**

43 patients with GPCC were screened. Only nine patients met inclusion criteria for QuickFISH™ testing. The average expected time avoided to optimize antibiotic therapy is 35 ± 16 hours. If the QuickFISH test had been indiscriminately implemented for all cases, the cost for performing this test would have been $5,590. However, using the prespecified algorithm, only 9 patients were tested for a projected cost of $1,170.

**Conclusion:**

Introducing rapid diagnostic testing in conjunction with implementing patient stratification algorithm for rapid identification of GPCC from blood cultures in addition to the ID pharmacy intervention will provide a positive impact on the clinical and economic outcomes in our health care setting.

## 1. Introduction

Coagulase-positive and coagulase-negative staphylococci (CoNS) are commonly isolated from blood cultures.* Staphylococcus aureus* (*S. aureus*) is responsible for a serious bacteremia requiring immediate antibiotic treatment. Delay in optimal therapy is associated with prolonged hospitalization and higher rates of mortality [1]. Vancomycin initiation is considered the standard of care for empiric therapy when Gram-positive cocci in clusters (GPCC) are reported in suspected blood stream infections. Furthermore, vancomycin continues to be first line for the treatment of methicillin-resistant* Staphylococcus aureus* (MRSA) bacteremia with minimum inhibitory concentrations of ≤1.5 mcg/mL. However, this agent has been found to be inferior to antistaphylococcal beta-lactams for methicillin-susceptible* Staphylococcus aureus* (MSSA) strains [2, 3]. A previous 6-month retrospective chart review was performed at our teaching hospital in an effort to characterize the most common Staph etiology. In this review, 16 cases were due to MSSA and 26 were due to MRSA. The time to optimal coverage for MSSA with an antistaphylococcal beta-lactam was 3.86 days. Patients with* S. aureus* bacteremia on average were hospitalized for 12.3 days and received 29.7 days of antibiotic therapy.

CoNS are common blood culture contaminants of which only about 20% are considered true opportunistic pathogens [4]. A typical contaminated blood culture investigation will reveal only one positive culture out of multiple bottle draws, no signs, or symptoms of endovascular infection and an immunocompetent host. However, true CoNS bloodstream infections are frequently associated with foreign bodies such as vascular catheters and implantable medical devices [5, 6]. Consequences of contaminated blood cultures include increased cost, prolonged hospitalization, overuse of antibiotics with associated adverse events, and additional work for members of the healthcare team [7]. A recent retrospective review at our teaching hospital identified a positive blood culture contamination rate of 35%.

Conventional methods of bacterial identification which differentiate MRSA from MSSA and CoNS typically require 24–48 hours from the time blood cultures turn positive [8]. Rapid diagnostics are capable of identifying these organisms in as little as 30 minutes from the positive blood culture report [6]. Several studies have demonstrated the following: a reduction in time to optimal antibiotic therapy by 1.7–2.5 days, a decrease in length of hospital stay by around 2–6 days, and a median reduction of hospital costs for up to $20,000 per patient [5, 7, 9]. Previous studies have also identified that a direct microbiology notification to an infectious disease (ID) pharmacy team results in facilitation of this intervention [6]. Patients with more than one infection pending microbiologic workup of other sites may require a continuation of empiric vancomycin and would therefore not be expected to maximally benefit from rapid blood culture identification.

There are several molecular FDA approved methods for rapid blood culture identification. Testing platforms include GeneXpert, MALDI TOF mass spectrometry, and QuickFISH. Molecular methods range in testing sophistication, need for equipment acquisition, and reagent cost. QuickFISH GPCC BC (QF GPCC) is a peptide nucleic acid fluorescence in situ hybridization (PNA-FISH) test capable of differentiating staphylococci in positive blood cultures. QuickFISH targets the 16S rRNA of* S. aureus* and CoNS directly from positive blood cultures with a sensitivity and specificity exceeding 98% [10, 11]. Fluorescently labeled probes bind to a specified region of the bacterial RNA to form a distinct green or red color when visualized under the microscope. Organism identification is based on color emission.

Our teaching hospital uses culture based technology to identify Staph species from positive blood cultures. The goal of this study is to evaluate whether introducing rapid diagnostic testing in conjunction with implementing a stratification algorithm for testing eligibility would be an appropriate clinical and cost saving approach.

## 2. Methodology

An internal concurrent 4-month observational study was performed. Due to platform availability at the time, QuickFISH was selected for this quality improvement project. For the purpose of performing this validation study, test kits and other equipment were provided by the manufacturer free of charge. This study was approved by the Hospital Integrity Board. During the study period, positive blood cultures continued to be worked up in accordance with the standard of care. An additional call to the ID pharmacy service occurred for all positive blood cultures with GPCC during the hours of 0700-1530. The ID pharmacy service investigated each case to determine if rapid identification of the* Staphylococcus* species would impact the use of antibiotics. If the ID pharmacy service deemed there is a need to continue empiric vancomycin, the rapid test would not be performed. However, if the Staph bacteremia was considered to be a monomicrobic infection or a potential contaminant and the patient was on vancomycin, the blood culture isolate would be rapidly identified.

**Figure 1:**
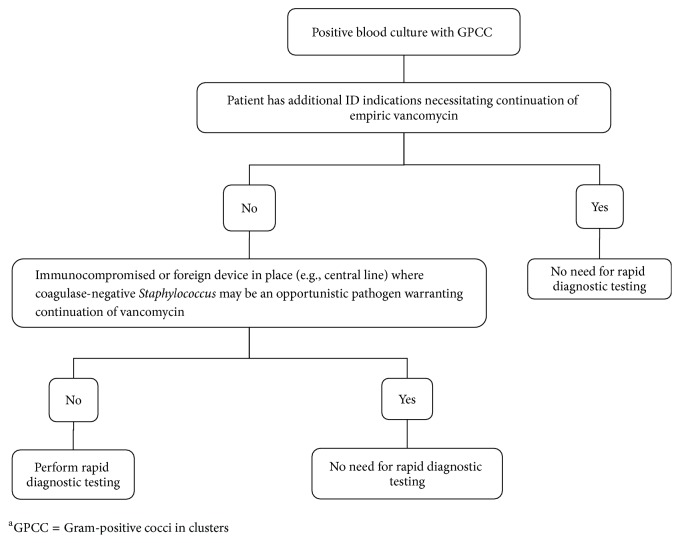
Criteria for rapid diagnostic testing^a^.

The target population for testing was identified using a stratified approach (Figure 1) and included age > 18 years, immunocompetent, no foreign devices in place such as central lines or cardiac devices, no additional infectious disease indications requiring continuation of empiric vancomycin, and no-risk factors for MRSA such as recent hospitalization, recent antibiotic exposure, prior MRSA infection, or nasal colonization. If the patient met criteria, the ID pharmacy service would notify microbiology to run the QuickFISH test. Results were not made available for patient intervention since the test results were used for research purposes only.

## 3. Definitions

We have defined optimal antibiotic therapy as the switch from empiric vancomycin to cefazolin or nafcillin in patients with MSSA bacteremia or discontinuation of unnecessary vancomycin in patients with CoNS if deemed to be a contaminant. Time to optimal antibiotic therapy for culture based methods was defined as the time microbiology reported the GPCC after performing the Gram stain to the time of antibiotic optimization. For rapid diagnostic testing, the expected time to optimal treatment for CoNS was defined as 2 hours from the time when GPCC was identified by Gram stain and 4 hours for* S. aureus* identification including mecA gene determination. We calculated the expected time that could have been avoided in hours to optimize antibiotic therapy using the new technology by subtracting 2 hours (if CoNS) or 4 hours (if MSSA) from the time to optimal antibiotic therapy using culture based methodology for microbiological identification.

## 4. Results

A total of 43 patients with GPCC were screened for QuickFISH test eligibility. Nine patients met the inclusion criteria for testing and therefore underwent QuickFISH (Table 1). Time to optimal antibiotic therapy may be decreased by approximately 20 to 74 hours (M = 35, SD = 16) for a total of 314 hours. Among the nine patients, 7 patients had CoNS and one had micrococcus. Among patients with CoNS, call back to the emergency department (ED) would have been avoided for 2 patients with contaminated bottles. One patient with MSSA bacteremia would have been expected to receive optimal therapy 74 hours earlier. A total of 34 patients did not meet the criteria for QuickFISH testing (Table 2). All the 34 patients received therapy with vancomycin for a range of 3–14 days (median = 6 days). The majority of these patients had CoNS (*N* = 28), 5 patients had MRSA, and 1 patient had MSSA. Of the 6 patients who were excluded due to presence of MRSA risk factors, 4 cultures were deemed to be contaminated and therefore we would have benefited from knowing the identification rapidly to prevent unnecessary antibiotic exposure. Projected hospital cost avoidance in the 9 cases that met the inclusion criteria for testing if rapid testing implemented is as follows: one patient with MSSA bacteremia: using primary literature cost data by Bauer and colleagues, the cost avoidance would have been $21,387; two ED patients with contaminated blood culture bottles: cost avoidance for ED call back would have been $718 × 2 = $1436 (this cost represents the ED visit only and does not include any interventions, doctors, pharmacists, and nurses time, medications, imaging studies, cultures, or laboratory tests); one patient had a contaminated blood culture bottle and discharge held by one day with a cost avoidance for extra day being approximately $1,024; and 5 patients for which vancomycin was initiated due to the panic call of GPCC in blood culture. The average drug acquisition cost of vancomycin per day is approximately $20. Therefore, $100 in drug acquisition cost could have been avoided (this cost does not include the cost for therapeutic drug monitoring or additional case workup for positive blood culture investigation). If we apply Gander and colleagues' estimates to all 8 contaminated blood cultures, the expected cost avoidance would total (8 × $8,720) $69,760 [12]. During the study period, 43 patients had GPCC reported in blood culture bottles. Additionally, the projected cost for performing this test is $130 per patient. This includes both the QuickFISH and mecA gene tests as well as labor cost. If the QuickFISH test had been indiscriminately implemented for all cases, the cost for performing this test would have been $5,590. However, using the stratified approach, only 9 patients were tested for a projected cost of $1,170 and the cost avoidance for untested patients, not including the 4 patients who were inappropriately excluded because of MRSA risk factors, would have been $3900.

## 5. Discussion

Time to optimal antibiotic therapy may be decreased by approximately 1–3 days if interventions have been made based on the results of the rapid diagnostic assays for the 9 cases that met the inclusion criteria for testing. Additionally, earlier discharge may be achieved and call back to the ED following discharge may be avoided. Stratification based on eligibility for the rapid identification test minimizes the use of this test for which early identification of GPCC is not likely to impact empiric antibiotic therapy. Therefore, a stratification algorithm for rapid molecular identification of GPCC from blood cultures was created accordingly (Figure 1). Among the 34 patients who did not meet the criteria for rapid testing, 30 patients were appropriately excluded, while the cultures for the remaining 4 patients were deemed to be contaminated and therefore we would have benefited from knowing the identification rapidly in order to prevent unnecessary antibiotic exposure. Therefore, we concluded that the presence of MRSA risk factor does not appear to be appropriate exclusion criteria and was consequently removed from the proposed stratification algorithm.

Even though molecular assays have been shown to reduce the time to optimal antibiotic therapy as well as length of hospital stay and cost of stay in previously published literature, in this observational study, we tested the appropriateness of a stratified approach to minimize the use of the rapid diagnostics to include only cases where early identification of GPCC is likely to impact empiric antibiotic therapy. However, this study has several limitations. First, it represented a small sample size because the ID PharmD is not available 24 hours per day for 7 days to receive all the notifications for GPCC. Second, it is a single center, 4-month observational trial. Third, rapid diagnostic was tested for validation only and the intervention was not allowed. Therefore, actual time to optimal antibiotic therapy for rapid diagnostic test was unable to be measured which could otherwise be longer than 2 hours for CoNS and 4 hours for* S. aureus*. This time included the expected microbiology workup time following the Gram stain and did not include the lag time between the microbiology reporting and the antibiotic optimization. A two-arm, prospective study design could overcome this limitation which is the potential future direction of this trial. Fourth, the presence of MRSA risk factors was not an appropriate exclusion criteria and was subsequently removed from the hospital's proposed rapid diagnostic stratification algorithm. Finally, some of the cost avoidance data were extrapolated from previously published cost avoidance trials.

## 6. Conclusion

Introducing rapid molecular testing in conjunction with implementing patient stratification algorithm for rapid molecular identification of GPCC from blood cultures in addition to the ID pharmacy intervention will provide a positive impact on the clinical and economic outcomes in our health care setting.

## Figures and Tables

**Table 1:** Patients who met inclusion criteria for rapid diagnostic testing^a^.

Age	Gender	Empiric therapy	Isolated microorganism	Plan made	Time to optimal antibiotic therapy (hours) using culture based method	Expected time avoided (hours) to optimal antibiotic therapy using the molecular diagnostic test
61	F	Vancomycin	MSSA	Changed to nafcillin	78	74
68	M	Vancomycin	CoNS	Vancomycin discontinued	44	42
32	F	Vancomycin and levofloxacin	CoNS	Discharged from ED	n/a	24^*∗*^
78	F	Vancomycin	CoNS	Vancomycin discontinued	30	28
74	F	Vancomycin and doxycycline	CoNS	Vancomycin discontinued and patient discharged	49	47
49	M	Vancomycin	CoNS	Discharged from ED	n/a	24^*∗*^
80	F	Vancomycin and ceftriaxone	Micrococcus	Vancomycin discontinued	28	26
85	F	Vancomycin and piperacillin/tazobactam	CoNS	Vancomycin discontinued	22	20
29	M	Vancomycin	CoNS	Vancomycin discontinued	31	29

^a^F = female, M = male, MSSA = methicillin-susceptible *Staphylococcus aureus*, CoNS = coagulase-negative staphylococci, and ED = emergency department. ^*∗*^Patients were called to return for emergency department evaluation, to assess clinical status and repeat blood cultures.

**Table 2:** Patients who did not meet inclusion criteria for rapid diagnostic testing^a^.

Reason for the exclusion	Number of patients (*n* = 34)
Additional infectious disease indications requiring continuation of empiric vancomycin	20
Immunocompromised host	3
Foreign device in place	5
Risk factor for MRSA	6

^a^MRSA = methicillin-resistant *Staphylococcus aureus*.
